# Replacement impacts of fish meal with corn protein concentrate in diets on growth, feed availability, and biochemical composition of rockfish (***Sebastes schlegeli***)

**DOI:** 10.1371/journal.pone.0322103

**Published:** 2025-04-29

**Authors:** Md. Farid Uz Zaman, Sung Hwoan Cho

**Affiliations:** 1 Department of Convergence Study on the Ocean Science and Technology, Korea Maritime and Ocean University, Busan, Republic of Korea; 2 Division of Convergence on Marine Science, Korea Maritime and Ocean University, Busan, Republic of Korea.; Universiti Malaysia Kelantan, MALAYSIA

## Abstract

Using fish meal (FM) as the predominant protein source in fish feeds for aquaculture operations poses considerable economic costs and ecological concerns. Therefore, feed nutritionists are looking for inexpensive and supply-stable alternatives to FM in fish feeds. This study aimed to assess the impacts of substituting FM with corn protein concentrate (CPC) in the diet of rockfish (*Sebastes schlegeli*) on the growth, feed availability, biochemical composition, and blood chemistry. Five hundred forty juvenile rockfish were uniformly dispersed into 18 tanks. Six isonitrogenous and isolipidic diets were prepared. The control (Con) diet contained 55% FM. The CPC_10_, CPC_20_, CPC_30_, CPC_40_, and CPC_50_ diets were prepared to substitute 10%, 20%, 30%, 40%, and 50% FM with CPC in the Con diet, respectively. Triplicate groups of rockfish received the experimental diets twice daily for 56 days. The weight gain (WG) of rockfish fed the Con and CPC_10_ diets was significantly (*P* < 0.001) greater than that of rockfish fed all other diets. Rockfish fed the Con and CPC_10_ diets exhibited a comparable specific growth rate (SGR) to that of fish fed the CPC_20_ diet. Feed consumption (FC) of rockfish fed the Con diet was significantly (*P* < 0.0001) higher than that of rockfish fed all other diets, with the exception of the CPC_10_ diet. Rockfish fed the Con, CPC_10_, and CPC_20_ diets exhibited significantly (*P* < 0.0001) higher feed efficiency (FE) compared to rockfish fed all other diets. Protein efficiency ratio of rockfish fed the Con diet was significantly (*P* < 0.001) higher than that of fish fed the CPC_40_ and CPC_50_ diets. Rockfish fed the Con diet exhibited significantly (*P* < 0.01) greater protein retention than rockfish fed the CPC_50_ diet. However, the biological indices except for hepatosomatic index, biochemical composition, and blood chemistry of rockfish were not significantly (*P* > 0.05) influenced by FM substitution with CPC in diets. Conclusively, up to 10% and 20% of FM can be substituted with CPC in the rockfish diet without significantly lowering WG and FC, and SGR and FE, respectively.

## Introduction

The need for fish meal (FM) in aquafeed has surged dramatically in recent decades because of the fast global expansion of the aquaculture industry. FM is commonly used as the predominant protein source in aquafeed due to its high protein content, balanced indispensable amino acid (AA), and fatty acid (FA) profiles, nutritional digestibility, minerals, and certain vitamins [1, 2]. However, due to the decline in marine fish populations, particularly forage fish stocks, along with the growing demand for FM driven by the intensification of aquaculture, the global market price of FM has been consistently rising [3, 4]. Therefore, feed nutritionists are increasingly seeking an affordable and sustainable substitute for FM.

In aquafeed, plant-derived protein alternatives have been extensively incorporated as replacements for FM [5–7] because of their cost-effectiveness, ready availability, high effectiveness, and environmental acceptability. Numerous investigations have been conducted to look for a replacer from plant-derived sources, such as grains, pulses, and oilseeds, for FM in the feeds of various carnivorous fish species [5]. The suitability of plant-derived protein sources as replacements for FM in aquafeed is often restricted due to some of the major constraints, such as unbalanced AA profiles and several anti-nutritional factors (ANFs), particularly trypsin inhibitors and phytic acid, which diminish the nutrient accessibility and digestibility of the feed [2,7–9]. However, several processing methods have been developed to ameliorate the nutritional profiles and minimize the ANFs, while raising the protein content of plant-based products [10, 11]. Various plant-derived proteins, including soybean, corn, canola, wheat, and pea have been used to produce protein concentrates. Plant protein concentrates, which are abundant in protein content but low in carbohydrate content, have been regarded as potential substitutes for FM particularly in high-protein feeds [12].

Corn protein concentrate (CPC), a newly produced form of corn protein, is predominantly derived from the endosperm by eliminating non-protein elements through enzymatic processing to solubilize proteins in wet milling operations [13, 14]. According to the Association of American Feed Control Officials (AAFCO) [15], upon refinement, CPC’s dry weight composition may include up to 80% crude protein and less than 1% starch. In addition, the use of CPC may reduce the need for additional methionine in diets, as it contains higher levels of this AA compared to other plant-derived feedstuffs [16–18]. However, CPC is often considered to be deficient in lysine content compared to soybean-based feedstuffs [2]. Several earlier studies have demonstrated that FM could be partially substituted with CPC without adversely compromising the growth and feed utilization of various fish species. For instance, Shekarabi et al. [19] unveiled that dietary substitution of 25.2% FM with CPC led to comparable growth performance to that of rainbow trout (*Oncorhynchus mykiss*) fed a diet containing 45.2% FM. Minjarez-Osorio et al. [17] also summarized that FM up to 50 and 75% could be substituted by CPC with AA supplementation in diets without impairing the growth of red drum (*Sciaenops ocellatus*) and shortfin corvina (*Cynoscion parvipinnis*), respectively. Furthermore, FM up to 50 and 53.4% could be substitutable by CPC supplemented with lysine in the red hybrid tilapia (*Oreochromis* sp.) and Nile tilapia (*Oreochromis niloticus*) diets, respectively, without any deleterious impacts on growth and feed utilization [10,20].

In the Republic of Korea (*hereafter,* Korea), rockfish (*Sebastes schlegeli*) is the second most extensively cultured marine finfish species, following olive flounder (*Paralichthys olivaceus*). Since the last few decades, it has been extensively cultured in Korea because of its fast growth, high disease resistance, and global market value [21]. The annual production of rockfish was 14,418 metric tons (MT) in 2023, which ranked the secondly highest out of all marine finfish aquaculture production of 79,651 MT in Korea [22]. Since rockfish, a carnivorous fish, has high dietary protein requirement [23], high quantity of FM is typically utilized as a predominant protein source in commercial rockfish diets [24], leading to high feed cost. Finding an alternative to FM in rockfish feed is essential for achieving sustainable and profitable rockfish production. To date, numerous researches have been conducted to assess the impact of replacing FM with different animal protein sources including tuna by-product meal and meat meal [25–28], plant protein sources including dehulled soybean meal (SBM), fermented SBM, and distillers dried grain [24,29,30], and their combinations including meat meal and corn gluten meal [31] on the growth performance of rockfish.

Substitutability of CPC for FM in diets may vary highly depending on fish species and/or AA supplementation that are likely to be deficient or lacking in CPC. Specifically, CPC supplemented with AA has been reported to replace FM up to 50% and 75% in the diets of various fish species [10,17,20]. However, for carnivorous species, such as rainbow trout, CPC without AA supplementation has been shown to replace FM up to 25.2% [19]. To our knowledge, there is a paucity of studies on using CPC without AA supplementation in the diets of rockfish. A study on the suitability of CPC in the diets of carnivorous fish species requiring relatively high levels of protein is needed prior to practical application. Therefore, the present study was designed to evaluate the impact of substituting FM with CPC on the growth, feed availability, biochemical composition, and blood chemistry of rockfish.

## Materials and methods

### Ethical Statement

All experimental procedures were reviewed and approved by the Institutional Animal Care and Use Committee (IACUC) of Korea Maritime and Ocean University, Busan, Republic of Korea (KMOU IACUC 2022-04) and were conducted in compliance with all applicable ethical regulations and guidelines. All research staff underwent comprehensive theoretical and practical training in animal care and handling prior to the study’s commencement, ensuring proper animal welfare and experimental integrity.

### Experimental design and feed formulation

A manufactured CPC known as Empyreal 75 (Cargill Corn Milling, Cargill Inc., Blair, NE, USA), which is rich in crude protein (79.7%) and abundant in methionine (1.77%), is commercially available and was used as an alternative protein source for FM in this study. Six isonitrogenous (51.0% crude protein, 8.16% nitrogen content) and isolipidic (12.5%) experimental feeds were formulated (Table 1). Specifically, the control (Con) diet was formulated to include 55% FM and 17.5% fermented SBM as the predominant protein sources. In the Con diet, 19% wheat flour was included as a carbohydrate source, while 3% each fish oil and soybean oil were incorporated as the lipid sources. The CPC_10_, CPC_20_, CPC_30_, CPC_40_, and CPC_50_ diets were prepared to substitute 10, 20, 30, 40, and 50% FM in the Con diet with CPC, respectively. Additionally, each experimental diet incorporated equal proportions of vitamin premix (1%), mineral premix (1%), and choline (0.5%). The experiment diets were prepared to fulfil the protein and lipid requirements for rockfish [32, 33]. All components of the feed were blended with water in a 3:1 proportion. The well-blended components were formed into pellets using a lab-scale extruder (Dongsung Mechanics, Busan, Korea) equipped with a 2-mm die hole plate. Subsequently, all the prepared feeds were dehydrated using an electronic drying unit (JW-1350ED; Jinwoo Electronics Co. Ltd., Hwaseong-Si, Gyeonggi-Do, Korea) at 40°C for 24 h. All experimental diets were sinking pellets. Finally, the experimental feeds were kept in a refrigerator at –20°C until further use.

**Table 1:** Ingredients and chemical composition of the experimental diets (%, dry matter basis; DM).

	Experimental diets					
	Con	CPC_10_	CPC_20_	CPC_30_	CPC_40_	CPC_50_
*Ingredients (%, DM)*						
Fish meal (FM)^1^	55.0	49.5	44.0	38.5	33.0	27.5
Corn protein concentrate (CPC)^2^		4.7	9.4	14.1	18.8	23.5
Fermented soybean meal^3^	17.5	17.5	17.5	17.5	17.5	17.5
Wheat flour	19.0	19.3	19.7	20.0	20.4	20.8
Fish oil	3.0	3.5	3.9	4.4	4.8	5.2
Soybean oil	3.0	3.0	3.0	3.0	3.0	3.0
Vitamin premix^4^	1.0	1.0	1.0	1.0	1.0	1.0
Mineral premix^5^	1.0	1.0	1.0	1.0	1.0	1.0
Choline	0.5	0.5	0.5	0.5	0.5	0.5
*Nutrients (%, DM)*						
Dry matter	98.4	98.4	98.1	98.3	98.3	97.8
Crude protein	50.9	50.9	51.2	51.2	50.7	51.3
Crude lipid	12.6	12.5	12.5	12.3	12.0	12.0
Ash	11.4	11.0	10.2	9.2	8.2	7.2
Carbohydrate^6^	25.1	25.6	26.1	27.3	29.1	29.5
Gross energy (kcal/g)^7^	4.2	4.2	4.2	4.2	4.3	4.3

^1^Fish meal (FM) (crude protein: 69.7%, crude lipid: 11.1%, ash: 16.7) was imported from Peru.

^2^Corn protein concentrate (CPC) (crude protein: 79.5%, crude lipid: 3.3%, ash: 1.0%) was imported from Cargill (Minneapolis, USA).

^3^Fermented soybean meal (crude protein: 53.2%, crude lipid: 1.6%, ash: 7.3%) was purchased from CJ CheilJedang (Seoul, Korea).

^4^Mineral premix contained the following ingredients (g/kg mix): MgSO_4_·7H_2_O, 80.0; NaH_2_PO_4_·2H_2_O, 370.0; KCl, 130.0; ferric citrate, 40.0; ZnSO_4_·7H_2_O, 20.0; Ca-lactate, 356.5; CuCl, 0.2; AlCl_3_·6H_2_O, 0.15; KI, 0.2; Na_2_Se_2_O_3_, 0.01; MnSO_4_·H_2_O, 2.0; CoCl_2_·6H_2_O, 1.0.

^5^Vitamin premix contained the following amount which were diluted in cellulose (g/kg mix): L-ascorbic acid, 121.2; DL-α-tocopheryl acetate, 75.7; thiamin hydrochloride, 2.7; riboflavin, 9.1; pyridoxine hydrochloride, 1.8; niacin, 36.4; Ca-D-pantothenate, 12.7; myo-inositol, 181.8; D-biotin, 5.45; folic acid, 0.68; p-aminobenzoic acid, 18.2; menadione, 1.8; retinyl acetate, 0.73; cholecalciferol, 0.003; cyanocobalamin, 0.003.

^6^Carbohydrate was calculated by the difference [carbohydrate = 100 – (crude protein + crude lipid + ash)].

^7^Gross energy (kcal/kg) was calculated by 4 kcal/g for protein and carbohydrate, and 9 kcal/g for lipid [34].

### Experimental fish and feeding trial

Healthy juvenile rockfish were procured from a commercial hatchery and underwent a 14-day acclimatization period before the start of the feeding experiment, during which fish received a commercial feed (50% crude protein and 8% crude lipid) manufactured by National Federation of Fisheries Cooperatives Feeds (Uiryeong-Gun, Gyeongsangnam-Do, Korea) administered twice daily. Total of 540 juvenile rockfish averaging 2.3 g were uniformly dispersed into 18, 50-L plastic tanks (30 juvenile/tank). All tanks were arranged with a proper aeration system throughout the experimental period, and continuously received a mixture of underground seawater and sand-filtered seawater (1:1) flowing at 4.2 L/min. The water quality was tracked on a daily basis over the experimental period using the AZ-8603 multi-parameter water quality instrument (AZ Instrument in Taichung, Taiwan). The parameters assessed were as follows: water temperature varied from 17.0 to 23.2°C (20.7 ± 1.53°C; mean ± SD), dissolved oxygen varied from 7.3 to 7.8 mg/L (7.5 ± 0.12 mg/L), salinity varied from 29.7 to 31.5 g/L (30.5 ± 0.41 g/L), and pH varied from 7.4 to 7.7 (7.5 ± 0.07). The photoperiod was not regulated, but adhered to the natural light-dark cycle. All experimental diets were administered to rockfish in triplicate groups and hand-fed twice a day (08:30 and 17:30) to achieve apparent satiation in feeding for 56 days. Fish health and behavior were carefully monitored twice daily throughout the experimental period to maintain the proper health of the rockfish. Siphon-cleaning was carried out daily to avoid the risk of deterioration of the water quality, and dead fish were promptly eliminated upon observation.

### Determination of growth, feed utilization, and biological indices of rockfish

At the termination of the 56-day feeding period, all surviving rockfish were fasted for 24 h. The number of live fish in each tank was recorded to measure survival, weight gain (WG), and specific growth rate (SGR) of rockfish. Fish were then randomly selected from each tank and carefully transferred using a knotless dip net to an anaesthetic bath prepared with 100 mg/L tricaine methanesulfonate (MS-222) for anaesthesia. An overdose of MS-222 was only used for euthanasia to minimize stress, pain, and discomfort throughout the process. Fish were monitored for anaesthesia by observing the loss of equilibrium and decreased movements, which typically occurred within 3–5 minutes, and they were euthanized within 10 minutes. Following that, ten anesthetized rockfish were randomly selected and underwent separate assessments of total length and weight to evaluate their condition factor (K). Subsequently, these ten fish were dissected, and the weights of their visceral and liver organs were recorded to assess the viscerosomatic index (VSI) and hepatosomatic index (HSI). Growth performance, feed availability, and biological metrics of rockfish were assessed using the following formulas: WG (g/fish) = final body weight of fish − initial body weight of fish, SGR (%/day) = [Ln final body weight of fish (g) − Ln initial body weight of fish (g)] × 100/days of feeding, feed consumption (FC, g/fish) = total feed consumption/number of surviving fish, feed efficiency (FE) = weight gain of fish/feed consumption, protein efficiency ratio (PER) = weight gain of fish/protein consumption, protein retention (PR, %) = protein gain of fish × 100/protein consumption, K (g/cm^3^) = fish weight (g) × 100/total length of fish (cm)^3^, VSI (%) = viscera weight of fish × 100/fish weight, and HSI (%) = liver weight of fish × 100/fish weight.

### Analysis of the biochemical composition of the experimental feeds and whole body of rockfish

Ten rockfish prior to the feeding experiment and the remaining rockfish (≥7) in each tank after the 56-day experimental period were subjected to homogenization and utilized for assessing the biochemical profiles of the whole body of rockfish. Following the Association of Official Analytical Chemists (AOAC) standard method [35], the chemical composition of the experimental diets and whole body of rockfish were analyzed. The Kjeldahl method, facilitated by the Kjeltec 2100, Distillation Unit from Foss Tecator (Hillerod, Denmark), was employed to ascertain the crude protein content. The crude lipid content was evaluated through an ether-extraction method, employing the Soxtec TM 2043 Fat Extraction System, manufactured by Foss Tecator (Hillerod, Denmark). For moisture content analysis, the samples underwent desiccation in an oven maintained at 105°C for a period of 24 for a duration of 24 h. The ash content was measured by subjecting the samples to a muffle furnace at 550°C for 4 h.

To analyze the AA, 18 components were measured using ion-exchange chromatography combined with the ninhydrin postcolumn reaction technique. The AA profiles of feed ingredients, experimental feeds, and whole-body rockfish were determined using the Hitachi L-8800 Auto-analyzer (Hitachi, Tokyo, Japan). In the analysis of 16 components (excluding methionine and cysteine), a 0.2 g sample was placed in a decomposition tube, mixed with 10 mL of 6 N HCl, purged with nitrogen gas, and then hydrolyzed at 110°C for 24 h. Following concentration with a reduced-pressure concentrator, the filtrate was brought to 50 mL with 0.2 M sodium citrate buffer and filtered through a 0.20-μm cellulose acetate syringe filter for use as the analysis sample. To analyze methionine and cysteine, samples underwent oxidation with performic acid at below 5°C for 24 h, resulting in methionine sulfone and cysteic acid. The samples were subsequently freeze-dried twice using deionized water, hydrolyzed, and analyzed in line with the standard analytical procedure used for other AAs. To assess the tryptophan content, the samples underwent analysis via high-performance liquid chromatography, utilizing an S1125 HPLC pump (Sykam, Germany). The analysis of the AA profiles of the samples followed the same protocols as those outlined in Lee et al. [27]’s study.

### Analysis of the blood chemistry of rockfish

Following the 56-day experimental period, rockfish were starved for 24 h. Fish were then randomly selected from each tank and anaesthetized with 100 mg/L tricaine methanesulfonate (MS-222). Blood specimens were drawn from ten anesthetized fish per tank via the caudal vein using 5 heparinized and 5 non-heparinized syringes to examine the plasma and serum parameters of rockfish, respectively. Plasma and serum were isolated through centrifugation (2,716 × *g* at 4°C) for 10 min, and promptly stored at −70°C in a freezer until analysis. Plasma was utilized to determine the aspartate aminotransferase (AST), alanine aminotransferase (ALT), alkaline phosphatase (ALP), total bilirubin (T-BIL), total cholesterol (T-CHO), triglyceride (TG), total protein (TP), albumin (ALB), and globulin (GLB) using an automated chemistry system (Fuji-Chem NX500i, Fujifilm, Tokyo, Japan). Serum was utilized in the assessment of superoxide dismutase (SOD) and lysozyme activity. The measurement of serum SOD was performed with an ELISA kit (MyBioSource, cat. No. MBS705758), following the supplier’s recommended procedures. A microplate reader (Tecan Infinite® 200 PRO, Zürich, Switzerland) was used to detect absorbance, and standard curves were plotted to calculate concentration. The procedure for the turbidimetric assay of lysozyme was executed in accordance with the study by Lange et al. [36]. Lysozyme analysis was carried out according to the same procedures and methods in Lee et al. [27]’s study.

### Statistical analysis

To ascertain significant variations between the dietary treatments, the data were subjected to one-way ANOVA and Tukey’s HSD post-hoc test using IBM SPSS Statistics version 24.0 (SPSS Inc., Chicago, IL, USA). Prior to performing ANOVA, tests for normality and homogeneity of variance were determined by the Shapiro-Wilk test and Levene’s test, respectively. Before conducting statistical analysis, all percentage data underwent arcsine transformation. Differences were deemed significant at *P* < 0.05. Orthogonal polynomial contrasts were applied to ascertain the response trends (linear, quadratic, or cubic) for each dependent variable versus dietary FM replacement levels with CPC. Regression analysis was then employed to determine the best-fitting model in cases where substantial differences were observed. Regression analysis was performed between WG, SGR, feed consumption, FER, PER, and CF of rockfish as the dependent variables and dietary FM replacement levels with CPC as the independent variable.

## Results

### AA profiles of the experimental diets

The essential AA (EAA) content, such as leucine, methionine, and phenylalanine in CPC was higher than that in FM, but lower for arginine, histidine, isoleucine, lysine, threonine, tryptophan, and valine content (). All of the non-essential AA (NEAA) content in FM were lower than that in CPC, except for aspartic acid and glycine. Leucine, methionine, and phenylalanine content appeared to rise as dietary FM substitution levels with CPC increased, but decrease for arginine, histidine, isoleucine, lysine, threonine, tryptophan, and valine content. All of the NEAA, except for aspartic acid and glycine content in the experimental diets were found to increase as dietary FM substitution levels with CPC increased.

### Growth performance, feed availability, and biological indices of rockfish

At the termination of the feeding experiment, the survival rate of rockfish varied from 94.4 to 97.8%, and was significantly (*P* > 0.1) unaffected by dietary FM substitution with CPC (Table 3). However, the WG of rockfish fed the Con and CPC_10_ diets was significantly (*P* < 0.0001) greater than that of rockfish fed all other diets. The SGR of rockfish fed the Con and CPC_10_ diets was also significantly (*P* < 0.0001) greater than that of rockfish fed the CPC_30_, CPC_40_, and CPC_50_ diets, but comparable to that of rockfish fed the CPC_20_ diet. In the context of the orthogonal polynomial contrast, WG and SGR of rockfish manifested significant (*P* = 0.0001 for both) linear relationships with dietary elevated FM replacement levels with CPC. Based on the regression analysis, the most appropriate models between dietary FM substitution levels with CPC and WG (Y = − 0.730000X + 10.0700, *P* < 0.0001, adjusted R^2^ = 0.9466) and SGR (Y = − 0.001300X + 0.0303, *P* < 0.0001, adjusted R^2^ = 0.8981) of rockfish were observed.

**Table 2:** Amino acid profiles (% of diet) of the fish meal (FM), corn protein concentrate (CPC), and experimental diets.

			Experimental diets						
	FM	CPC	Requirement	Con	CPC_10_	CPC_20_	CPC_30_	CPC_40_	CPC_50_
*Essential amino acids (EAA, %)*									
Arginine	3.51	1.99		2.61	2.57	2.48	2.37	2.29	2.26
Histidine	1.50	1.35		1.03	1.03	1.00	0.99	0.96	0.93
Isoleucine	2.47	2.07		1.70	1.67	1.66	1.63	1.59	1.55
Leucine	4.50	12.70		3.59	3.73	3.98	4.31	4.71	5.02
Lysine	4.82	0.93	2.99^1^	3.13	3.07	3.00	2.75	2.49	2.22
Methionine	1.75	1.80	1.37^2^	1.13	1.15	1.16	1.17	1.24	1.24
Phenylalanine	2.42	4.48		1.97	2.03	2.14	2.26	2.34	2.47
Threonine	2.69	2.44		2.00	1.97	1.91	1.91	1.88	1.87
Tryptophan	0.49	0.29		0.33	0.35	0.30	0.31	0.28	0.27
Valine	2.96	2.59		2.01	1.98	1.97	1.97	1.96	1.96
**∑**EAA^3^	27.11	30.64		19.50	19.55	19.60	19.67	19.74	19.79
*Non-essential amino acids (NEAA, %)*									
Alanine	4.04	5.67		2.88	2.90	2.93	3.02	3.07	3.12
Aspartic acid	5.58	3.74		4.33	4.26	4.05	3.90	3.84	3.73
Cysteine	0.80	1.70	0.12^2^	0.69	0.73	0.78	0.82	0.83	0.88
Glutamic acid	8.08	13.80		7.15	7.25	7.38	7.52	7.62	7.69
Glycine	3.94	1.68		2.72	2.61	2.47	2.26	2.11	2.03
Proline	2.65	6.47		2.39	2.54	2.64	2.76	2.83	2.90
Serine	2.46	3.26		2.08	2.10	2.13	2.17	2.21	2.25
Tyrosine	1.53	2.84		1.19	1.22	1.26	1.31	1.34	1.38
**∑**NEAA^4^	29.08	39.16		23.43	23.61	23.64	23.76	23.85	23.98

^1^Data were obtained from Yan et al. [37]’s study.

^2^Data were obtained from Yan et al. [38]’s study.

^3^∑EAA: Total essential amino acids.

^4^∑NEAA: Total non-essential amino acids.

**Table 3:** Survival (%), weight gain (WG, g/fish), specific growth rate (SGR, %/day), feed consumption (FC, g/fish), feed efficiency (FE), protein efficiency ratio (PER), protein retention (PR, %), condition factor (K, g/cm^3^), viscerosomatic index (VSI, %), and hepatosomatic index (HSI, %) of rockfish fed the experimental diets for 56 days.

	Experimental diets							Orthogonal polynomial contrast			Regression analysis		
	Con	CPC_10_	CPC_20_	CPC_30_	CPC_40_	CPC_50_	*P*-value	Linear	Quadratic	Cubic	Equation	*P*-value	Adj. R^2^
IBW (g/fish)	2.4 ± 0.02	2.4 ± 0.02	2.4 ± 0.02	2.3 ± 0.00	2.3 ± 0.00	2.3 ± 0.00	*P* > 0.3						
FBW (g/fish)	12.6 ± 0.25^a^	11.8 ± 0.26^a^	10.8 ± 0.11^b^	10.2 ± 0.19^b^	9.4 ± 0.10^c^	8.9 ± 0.07^c^	*P* < 0.0001	0.0001	0.3775	0.6144	Y = − 0.759048X + 13.2844	*P* < 0.0001	0.9521
Survival (%)	95.6 ± 1.11	97.8 ± 1.11	97.8 ± 1.11	94.4 ± 1.11	94.4 ± 1.11	95.6 ± 1.11	*P* > 0.1						
WG (g/fish)	10.2 ± 0.28^a^	9.5 ± 0.28^a^	8.5 ± 0.13^b^	7.9 ± 0.19^bc^	7.0 ± 0.10 cd	6.5 ± 0.07^d^	*P* < 0.0001	0.0001	0.4017	0.6685	Y = − 0.730000X + 10.0700	*P* < 0.0001	0.9466
SGR (%/day)^1^	2.97 ± 0.053^a^	2.88 ± 0.055^a^	2.72 ± 0.035^ab^	2.64 ± 0.034^bc^	2.48 ± 0.019 cd	2.38 ± 0.014^d^	*P* < 0.0001	0.0001	0.5117	0.3191	Y = − 0.001300X + 0.0303	*P* < 0.0001	0.8981
FC (g/fish)^2^	9.70 ± 0.182^a^	9.25 ± 0.417^ab^	8.38 ± 0.060^bc^	8.20 ± 0.284^bc^	7.60 ± 0.095^c^	7.36 ± 0.056^c^	*P* < 0.0001	0.0001	0.2236	0.8713	Y = − 0.455000X + 9.5230	*P* < 0.0001	0.8359
FE^3^	1.04 ± 0.007^a^	1.03 ± 0.007^a^	1.00 ± 0.006^a^	0.95 ± 0.014^b^	0.91 ± 0.007^c^	0.87 ± 0.008^c^	*P* < 0.0001	0.0001	0.0266	0.0326	Y = − 0.036124X + 1.0936	*P* < 0.0001	0.9230
PER^4^	2.06 ± 0.018^a^	2.02 ± 0.052^ab^	1.97 ± 0.017^ab^	1.89 ± 0.024^abc^	1.82 ± 0.017^bc^	1.73 ± 0.022^c^	*P* < 0.001	0.0001	0.3633	0.8994	Y = − 0.000600X + 0.0213	*P* < 0.0001	0.7425
PR (%)^5^	32.3 ± 1.476^a^	31.7 ± 0.999^ab^	31.4 ± 0.618^ab^	28.8 ± 0.864^ab^	28.7 ± 0.263^ab^	27.3 ± 0.897^b^	*P* < 0.01	0.001	0.7045	0.6863	Y = − 0.010099X + 0.3424	*P* < 0.0001	0.5116
K (g/cm^3^)^6^	1.55 ± 0.029	1.49 ± 0.008	1.52 ± 0.014	1.51 ± 0.029	1.49 ± 0.021	1.45 ± 0.014	*P* > 0.1	0.0638	0.6042	0.4554			
VSI (%)^7^	9.44 ± 0.126	9.60 ± 0.131	9.63 ± 0.192	10.02 ± 0.400	10.14 ± 0.349	10.28 ± 0.198	*P* > 0.2	0.1364	0.4111	0.2363			
HSI (%)^8^	2.17 ± 0.118^b^	2.37 ± 0.108^ab^	2.61 ± 0.116^ab^	3.04 ± 0.32^ab^	3.13 ± 0.328^a^	3.20 ± 0.068^a^	*P* < 0.01	0.001	0.4732	0.5102	Y = 0.002238X + 0.0198	*P* < 0.0001	0.5929

Values (means of triplicate ± SE) in the same column that share the same superscript letter are not significantly different (*P* > 0.05).

Abbreviations: IBW, initial body weight; FBW, final body weight; Adj.R^2^, adjusted R square.

^1^Specific growth rate (SGR, %/day) = [Ln final body weight of fish (g) – Ln initial body weight of fish (g)] × 100/days of feeding.

^2^Feed consumption (FC, g/fish) = total feed consumption/number of surviving fish.

^3^Feed efficiency (FE) = weight gain of fish/feed consumption.

^4^Protein efficiency ratio (PER) = weight gain of fish/protein consumption.

^5^Protein retention (PR, %) = protein gain of fish × 100/protein consumption.

^6^Condition factor (K, g/cm^3^) = fish weight (g) × 100/total length of fish (cm)^3^.

^7^Viscerosomatic index (VSI, %) = viscera weight of fish × 100/fish weight.

^8^Hepatosomatic index (HSI, %) = liver weight of fish × 100/fish weight.

The FC of rockfish fed the Con diet was significantly (*P* < 0.0001) greater than that of rockfish fed all other diets, with the exception of the CPC10 diet (Table 3). Based on the orthogonal polynomial contrast, FC exhibited a significant (*P =* 0.0001) linear relationship with rising levels of FM substitution by CPC in diets. A linear model (Y = − 0.455000X + 9.5230, *P* < 0.0001, adjusted R^2^ = 0.8359) was found to be the most appropriate for describing the relationship between FM replacement levels in the diets with CPC and FC in regression analysis.

The FE of rockfish fed the Con, CPC_10_, and CPC_20_ diets was significantly (*P* < 0.0001) greater than that of rockfish fed all other diets. In the orthogonal polynomial contrast, the FE of rockfish exhibited significant linear (*P* = 0.0001), quadratic (*P* = 0.0266), and cubic (*P* = 0.0326) relationships with dietary elevated FM substitution levels by CPC. A linear model (Y = − 0.036124X + 1.0936, *P* < 0.0001, adjusted R^2^ = 0.9230) was observed as the most appropriate for describing the relationship between dietary FM replacement levels with CPC and FE in the regression analysis.

Rockfish fed the Con diet showed significantly (*P* < 0.001) greater PER than that fed the CPC_40_ and CPC_50_ diets, but no significant (*P* > 0.05) difference was observed compared to that fed the CPC_10_, CPC_20_, and CPC_30_ diets. The PR of rockfish fed the Con diet was significantly (*P* < 0.01) greater than that of rockfish fed the CPC_50_ diet, but not significantly (*P* > 0.05) different from that of rockfish fed the CPC_10_, CPC_20_, CPC_30_, and CPC_40_ diets. In regarding the orthogonal polynomial contrast, PER and PR of rockfish manifested significant (*P* = 0.0001 and *P* = 0.001, respectively) linear relationships with dietary elevated FM substitution levels with CPC. Based on the regression analysis, the most appropriate models between dietary elevated FM substitution levels with CPC and PER (Y = − 0.000600X + 0.0212, *P* < 0.0001, adjusted R^2^ = 0.7425), and PR (Y = − 0.010099X + 0.3424, *P* < 0.0001, adjusted R^2^ = 0.5116) of rockfish were observed.

The K and VSI of rockfish were not significantly (*P* > 0.05) altered by dietary CPC substitution for FM. However, HSI of rockfish fed the CPC_40_ and CPC_50_ diets was significantly (*P* < 0.01) greater than that of rockfish fed the Con diet, but not significantly (*P* > 0.05) different from that of rockfish fed the CPC_10_, CPC_20_, and CPC_30_ diets. The orthogonal polynomial contrast revealed that the HSI of rockfish exhibited a significant (*P* = 0.001) linear relationship with increasing dietary FM substitution levels with CPC. Based on the regression analysis, a linear relationship between dietary FM substitution levels with CPC and HSI (Y = 0.002238X + 0.0198, *P* < 0.0001, adjusted R^2^ = 0.5929) of rockfish was observed.

### Biochemical composition of the whole body of rockfish

The whole-body proximate composition of rockfish showed variations in moisture (72.8–73.3%), crude protein (15.3–15.8%), crude lipid (6.9–7.2%), and ash content (3.5–4.3%) (Table 4). None of these parameters significantly (*P* > 0.05) differed by dietary FM substitution with CPC.

**Table 4:** Proximate composition (% of wet weight) of rockfish fed the experimental diets for 56 days.

	Experimental diets							Orthogonal polynomial contrast		
	Con	CPC_10_	CPC_20_	CPC_30_	CPC_40_	CPC_50_	*P*-value	Linear	Quadratic	Cubic
Moisture	73.2 ± 0.07	73.0 ± 0.72	73.1 ± 0.23	73.3 ± 0.31	73.0 ± 0.54	72.8 ± 0.44	*P* > 0.9	0.5941	0.6904	0.6374
Crude protein	15.6 ± 0.64	15.6 ± 0.25	15.8 ± 0.35	15.3 ± 0.27	15.6 ± 0.20	15.6 ± 0.52	*P* > 0.9	0.9463	0.8332	0.7286
Crude lipid	6.9 ± 0.15	7.2 ± 0.17	7.1 ± 0.26	6.9 ± 0.35	7.0 ± 0.06	7.0 ± 0.15	*P* > 0.8	0.8081	0.5420	0.2947
Ash	4.3 ± 0.27	4.2 ± 0.23	3.7 ± 0.28	3.7 ± 0.20	3.6 ± 0.15	3.5 ± 0.09	*P* > 0.07	0.0624	0.3162	0.8567

Values are presented as means of triplicate ± SE.

The AA profiles of the whole body of rockfish were not significantly (*P* > 0.05) affected by dietary FM replacement with CPC (Table 5).

**Table 5:** Amino acid (% of wet weight) compositions of the whole body of rockfish fed the experimental diets for 56 days.

	Experimental diets							Orthogonal polynomial contrast		
	Con	CPC_10_	CPC_20_	CPC_30_	CPC_40_	CPC_50_	*P*-value	Linear	Quadratic	Cubic
*Essential amino acid (%)*										
Arginine	0.94 ± 0.029	0.99 ± 0.032	0.91 ± 0.020	0.91 ± 0.032	0.94 ± 0.023	0.94 ± 0.020	*P >* 0.3	0.4243	0.4971	0.3020
Histidine	0.32 ± 0.020	0.34 ± 0.026	0.32 ± 0.032	0.33 ± 0.017	0.33 ± 0.026	0.32 ± 0.015	*P >* 0.9	0.8546	0.9152	0.8435
Isoleucine	0.58 ± 0.046	0.59 ± 0.026	0.57 ± 0.035	0.58 ± 0.038	0.58 ± 0.026	0.57 ± 0.040	*P >* 0.9	0.8791	0.9209	0.8708
Leucine	1.08 ± 0.046	1.10 ± 0.029	1.07 ± 0.038	1.07 ± 0.043	1.09 ± 0.026	1.08 ± 0.043	*P >* 0.9	0.9269	0.9333	0.9949
Lysine	1.20 ± 0.029	1.24 ± 0.035	1.18 ± 0.015	1.20 ± 0.029	1.20 ± 0.035	1.19 ± 0.023	*P >* 0.7	0.5474	0.9899	0.7252
Phenylalanine	0.57 ± 0.038	0.59 ± 0.032	0.57 ± 0.043	0.56 ± 0.038	0.57 ± 0.043	0.56 ± 0.035	*P >* 0.9	0.7990	0.8379	0.8488
Threonine	0.70 ± 0.038	0.71 ± 0.040	0.69 ± 0.038	0.68 ± 0.049	0.70 ± 0.035	0.70 ± 0.038	*P >* 0.9	0.9889	0.7678	0.8304
Tryptophan	0.10 ± 0.046	0.05 ± 0.015	0.05 ± 0.020	0.05 ± 0.023	0.05 ± 0.003	0.04 ± 0.017	*P >* 0.5	0.1482	0.3607	0.4329
Valine	0.67 ± 0.061	0.68 ± 0.023	0.66 ± 0.043	0.66 ± 0.035	0.67 ± 0.035	0.66 ± 0.038	*P >* 0.9	0.8253	0.8608	0.9904
*Non-essential amino acid (%)*										
Alanine	1.03 ± 0.049	1.08 ± 0.015	0.99 ± 0.026	0.99 ± 0.052	1.06 ± 0.035	1.04 ± 0.035	*P >* 0.5	0.9667	0.4573	0.7020
Aspartic acid	1.47 ± 0.043	1.51 ± 0.043	1.43 ± 0.029	1.43 ± 0.032	1.48 ± 0.035	1.46 ± 0.035	*P >* 0.5	0.5881	0.4752	0.7197
Glutamic acid	2.03 ± 0.058	2.01 ± 0.026	1.99 ± 0.032	1.96 ± 0.032	2.05 ± 0.043	2.04 ± 0.038	*P >* 0.6	0.7296	0.2086	0.8531
Glycine	1.20 ± 0.058	1.31 ± 0.038	1.10 ± 0.061	1.17 ± 0.061	1.22 ± 0.032	1.19 ± 0.026	*P >* 0.1	0.6045	0.4670	0.6820
Proline	0.69 ± 0.052	0.75 ± 0.029	0.66 ± 0.038	0.66 ± 0.038	0.71 ± 0.046	0.70 ± 0.049	*P >* 0.6	0.7979	0.6295	0.5631
Serine	0.72 ± 0.049	0.75 ± 0.029	0.69 ± 0.032	0.68 ± 0.040	0.72 ± 0.032	0.71 ± 0.029	*P >* 0.8	0.6341	0.6079	0.6085
Tyrosine	0.40 ± 0.029	0.42 ± 0.072	0.39 ± 0.040	0.42 ± 0.046	0.40 ± 0.035	0.40 ± 0.043	*P >* 0.9	0.9530	0.9143	0.9247

Values are presented as means of triplicate ± SE.

### Blood chemistry measurements of rockfish

The plasma AST of rockfish varied from 146.7 to 152.0 IU/L, ALT varied from 23.0 to 24.3 IU/L, ALP varied from 182.3 to 186.7 IU/L, T-BIL varied from 1.5 to 1.8 mg/dL, T-CHO varied from 250.0 to 254.3 mg/dL, TG varied from 358.0 to 363.3 mg/dL, TP varied from 4.2 to 4.6 g/dL, ALB varied from 1.1 to 1.4 g/dL, and GLB varied from 3.0 to 3.5 g/dL (Table 6). None of the plasma parameters measured in rockfish were found to be significantly (*P* > 0.05) influenced by substituting FM with CPC in the diet.

The serum SOD varied from 2.3 to 2.5 ng/mL, and the lysozyme activity of rockfish varied from 336.5 to 446.0 U/mL. No significant (*P >* 0.05) alterations were detected in these parameters by dietary FM replacement with CPC.

**Table 6:** Blood chemistry of rockfish fed the experimental diets for 56 days.

	Experimental diets							Orthogonal polynomial contrast		
	Con	CPC_10_	CPC_20_	CPC_30_	CPC_40_	CPC_50_	*P*-value	Linear	Quadratic	Cubic
*Plasma parameters*										
AST (IU/L)	150.7 ± 0.73	150.0 ± 1.80	152.0 ± 0.87	149.0 ± 0.76	146.7 ± 0.93	149.0 ± 0.58	*P* > 0.5	0.2373	0.9032	0.3456
ALT (IU/L)	24.3 ± 0.73	24.0 ± 0.58	23.7 ± 0.60	23.7 ± 0.44	23.0 ± 0.87	23.7 ± 0.88	*P* > 0.9	0.5988	0.7800	0.8486
ALP (IU/L)	184.3 ± 0.88	185.0 ± 0.50	183.7 ± 0.44	186.7 ± 0.60	182.3 ± 0.73	186.3 ± 0.60	*P* > 0.2	0.6499	0.6986	0.3523
T-BIL (mg/dL)	1.8 ± 0.23	1.7 ± 0.10	1.5 ± 0.12	1.7 ± 0.13	1.6 ± 0.21	1.7 ± 0.11	*P* > 0.9	0.7855	0.6444	0.8349
T-CHO (mg/dL)	253.3 ± 0.88	254.3 ± 0.73	253.3 ± 0.73	250.0 ± 0.87	253.7 ± 0.60	252.3 ± 0.93	*P* > 0.5	0.4531	0.6401	0.5543
TG (mg/dL)	363.3 ± 0.88	363.0 ± 0.76	358.0 ± 0.76	362.7 ± 0.73	359.3 ± 0.67	359.7 ± 0.93	*P* > 0.1	0.0879	0.5048	0.6042
TP (g/dL)	4.6 ± 0.17	4.4 ± 0.08	4.5 ± 0.13	4.2 ± 0.23	4.5 ± 0.15	4.5 ± 0.28	*P* > 0.9	0.8326	0.5845	0.9736
ALB (g/dL)	1.2 ± 0.09	1.3 ± 0.13	1.1 ± 0.12	1.2 ± 0.10	1.4 ± 0.15	1.2 ± 0.11	*P* > 0.9	0.7287	0.9157	0.8446
GLB (g/dL)	3.5 ± 0.09	3.1 ± 0.18	3.4 ± 0.03	3.0 ± 0.16	3.1 ± 0.24	3.3 ± 0.21	*P* > 0.9	0.6326	0.5950	0.7285
*Serum parameters*										
SOD (ng/mL)	2.5 ± 0.13	2.4 ± 0.10	2.3 ± 0.04	2.4 ± 0.15	2.5 ± 0.08	2.5 ± 0.07	*P* > 0.9	0.8736	0.4510	0.6681
Lysozyme activity (U/mL)	399.9 ± 36.0	369.2 ± 28.9	336.5 ± 12.7	362.4 ± 43.2	446.0 ± 48.3	373.7 ± 88.5	*P* > 0.7	0.7640	0.5766	0.2615

Values are presented as means of triplicate ± SE.

Abbreviations: AST, Aspartate aminotransferase; ALT, alanine aminotransferase; ALP, alkaline phosphatase; T-BIL, total bilirubin; T-CHO, total cholesterol; TG, triglyceride; TP, total protein; ALB, albumin; GLB, globulin; and SOD, superoxide dismutase.

## Discussion

Developing plant-based alternatives to FM can be an effective approach to achieve sustainable and economically viable aquaculture, addressing the rising global seafood demand, while simultaneously mitigating the depletion of marine fish resources [5]. This study aimed to assess the suitability of CPC as a viable plant-based substitute for FM in the diets of rockfish. No discernible differences in WG and FC of rockfish fed the Con and CPC_10_ diets implied that 10% FM can be substituted with CPC in the 55% FM-based diet of rockfish without deteriorating WG and FC. Furthermore, no difference in SGR of rockfish fed the Con and CPC_20_ diets also suggested that FM up to 20% can be substitutable with CPC in the 55% FM-based diet without lowering the SGR of rockfish. As SGR values (2.38–2.97%/day) of rockfish in this experiment were comparable to or higher than those previously reported in rockfish with similar sizes (1.97–3.23, 1.89–2.40, 2.64–2.93, 2.63–3.01, and 2.37–2.64%/day for the initial weights of 2.5, 3.2, 2.3, 2.4, and 3.6 g, respectively) in other studies [24,25,27,28,31], the growth performance of rockfish was comparatively well achieved. The outcomes of the present experiment are in agreement with Shekarabi et al. [19]’s study, in which the replacement of FM up to 25.2% with CPC in the diet did not exhibit any discernible change in growth performance in comparison to rainbow trout fed a 45.2% FM-based diet. However, further FM substitution led to inferior growth performance when fish were provided with a 45% FM-based diet or one of diets replacing 8.3, 16.8, 25.2, and 33.7% FM with CPC for 56 days. They estimated the optimum substitution level of FM with CPC to be 8.1–8.2% in diet, as determined by the broken-line model analysis of SGR and FE. However, some other fish species have exhibited relatively high tolerance to dietary substitution of FM by CPC with AA supplementation. For instance, Ng et al. [10] confirmed that FM up to 50% could be substituted by CPC supplemented with lysine in the diet without retarding the growth of red hybrid tilapia. Likewise, Minjarez-Osorio et al. [17] unveiled that FM replacement up to 50% could be made by CPC fortified with crystalline AA in the diet without deteriorating the growth performance of red drum. Furthermore, the substitution of FM up to 53.4% by CPC supplemented with lysine in the diet did not considerably hinder the growth of Nile tilapia [20]. Considering the results of relatively high FM replacement levels with CPC in these studies [10,17,20] compared to the 10–20% FM substitution with CPC in this study, the substitutability of FM with CPC can vary depending on fish species, fish size, and the presence or absence of AA supplementation, particularly lysine, which is likely to be deficient in CPC.

The WG, SGR, and FC of rockfish linearly declined as the dietary FM substitution levels with CPC elevated in regression analysis. The linear reduction in FC with high FM replacement levels by CPC in this experiment could be elucidated by the studies of Carr et al. [39] and Men et al. [40], which indicate that plant-based protein sources are often lack water-soluble biochemical compounds responsible for diet’s palatability. Furthermore, Ng et al. [41] revealed that the inclusion of 0.5% betaine and 2% dried basil leaves as palatability enhancers in the diet of red hybrid tilapia significantly improved growth performance and feed utilization, as reflected in improved feed intake and nutrient digestibility, when substituting FM up to 75% with a blend of CPC and soy protein concentrate (SPC) at 2:1. Therefore, incorporating a feed palatability enhancer to develop low FM diets for desirable and sustainable fish culture could be applicable in future studies.

The shortage of EAA is widely regarded as a bottleneck to incorporate an alternative protein source in fish feed formulations [42]. Therefore, the AA profiles in low FM diets for fish should be carefully considered because deficiencies of some EAAs may often result in poor fish growth [30,43,44]. According to Tian et al. [45], a lack of one or more EAAs in diets commonly prevents other AAs from being absorbed and makes it or them more likely to be utilized as the energy source. Lysine and methionine are well known as the limiting EAAs in the majority of feed ingredients, in particular, those derived from plants [46, 47]. A deficiency of lysine in diets resulted in low feed utilization and stunted growth in rainbow trout (*Oncorhynchus mykiss*) [48], grass carp (*Ctenopharyngodon idellus*) [49], and seabass (*Lateolabrax japonicus*) [50]. Nonetheless, a number of variables, including fish size (age), sex, water temperature, and feed composition, may influence the EAA requirements of fish [44]. In the current experiment, the Con, CPC_10_, and CPC_20_ diets met the dietary lysine requirement for rockfish [37], which could elucidate why rockfish fed these diets showed superior growth performance (WG or SGR) in comparison to those fed the CPC_30_, CPC_40,_ and CPC_50_ diets. Therefore, there might be potential to enhance the incorporation levels of CPC for dietary FM replacement by supplementing particular limiting AAs, such as lysine that are deficient in CPC. Nevertheless, all experimental diets, including the Con diet, had slightly lower methionine content than the dietary methionine requirement of 1.37% in the presence of 0.12% cysteine for rockfish [38]. Interestingly, the cysteine content in all experimental diets varied from 0.69% in the Con diet to 0.88% in the CPC_50_ diet in the present experiment, which was higher than the cysteine content (0.12%) in Yan et al. [38]’s study. As the cysteine is capable of sparing 40, and 50% of the methionine requirements in the diets of stinging catfish (*Heteropneustes fossilis*) and red drum, respectively [51, 52], higher cysteine content might lower dietary methionine requirement of rockfish in this experiment.

Determining nutrient digestibility through digestibility studies is important to evaluate the efficiency of feedstuffs and the proper use of nutrients in animal feed formulations [53]. Digestibility of CPC by rockfish had not determined in this study. To the best of our knowledge, no study has been reported on the digestibility of CPC in carnivorous fish species, except for red hybrid tilapia (*Oreochromis* sp.) in Ng et al. [10]’ study, in which higher substitution of FM with CPC in diets led to lower digestibility of nutrients in red hybrid tilapia. In general, the digestibility coefficients of FM and plant protein sources in diets might differ among fish species, with FM generally exhibiting higher digestibility [5,54]. Incorporating high levels of plant sources into the diets of Asian seabass might lead to decreased digestibility because of an imbalanced EAA profile [55]. The digestibility of feeds affected the availability of EAA by fish, as higher digestibility led to higher nutrient absorption and utilization [54,56]. In addition, elevated inclusion levels of corn-based protein, such as CPC and corn gluten meal in diets led to lower digestibility of nutrients in red hybrid tilapia [10], and Asian seabass (*Lates calcarifer*) [55] and turbot (*Psetta maxima*) [56], respectively.

Superior FE was observed in rockfish fed the Con, CPC_10_, and CPC_20_ diets in comparison to fish fed the CPC_30_, CPC_40_, and CPC_50_ diets. The PER and PR of rockfish fed the Con diet were comparable to rockfish fed the CPC_10_, CPC_20_, and CPC_30_ diets and rockfish fed the CPC_10_, CPC_20,_ CPC_30_, and CPC_40_ diets, respectively. FE, PER, and PR of rockfish linearly declined with increased FM substitution levels with CPC in this experiment. This finding is congruent with the earlier research reported by Minjarez-Osorio et al. [17], who revealed that dietary elevated substitution levels of CPC, SBM, and soy protein concentrate supplemented with AA for FM lowered feed utilization of red drum and shortfin corvina. Furthermore, Lim et al. [24] also stated that FE and PR of rockfish tended to decline with dietary elevated FM substitution levels with dehulled SBM regardless of AA supplementation.

The biological indices, such as K, VSI, and HSI, are often employed as indicators to assess the nutritional status and reveal the physiological conditions of fish [57]. Specifically, K is considered a marker that indicates the level of energy reserves in fish, with fluctuations in K signalling changes in health and nutritional status [58, 59]. The VSI and HIS, on the other hand, are linked to the lipid levels in the viscera and liver, which may influence the yield in fish production [60]. The K and VSI of rockfish were not altered by dietary substitution of CPC for FM in this experiment. Likewise, earlier studies reported no discernible alterations in K [19,55,61–63] and VSI [10,55,61,63] of different fish species when various levels of dietary plant-origin alternatives were substituted for FM. However, the HSI of rockfish fed the CPC_40_ and CPC_50_ diets was markedly higher than that of fish fed the Con diet. In addition, the HSI of rockfish linearly increased with dietary FM substitution with CPC. The relatively high carbohydrate levels in all CPC diets (25.6–29.5%) compared to the Con (25.1%) diet could lead to metabolic disturbances and increased glycogen storage in the liver, ultimately contributing to liver enlargement and an elevated HSI of rockfish in this experiment. Likewise, Ye et al. [62] and Seong et al. [64] demonstrated that increased dietary FM replacement with SBM and fermented plant-based protein concentrate significantly elevated HSI of olive flounder. Similar outcomes regarding the HSI of red hybrid tilapia [10] and rockfish [24,65] were also demonstrated. In contrast to the finding of this experiment, Lim et al. [66] observed a downward trend in the HSI of tiger puffer (*Takifugu rubripes*) as dietary SBM substitution for FM increased.

No remarkable alteration was observed in the proximate composition of the whole-body rockfish in this experiment. This result is in accordance with the prior findings, which demonstrated no considerable alterations in the chemical composition of hybrid grouper fed diets substituting FM with soy protein concentrate (SPC) [67], and olive flounder [62] and Nile tilapia [68] fed diets substituting FM with SBM. Furthermore, replacing FM with blended SBM, corn gluten meal (CGM), and CPC, or with blended SBM, canola meal, and cotton seed meal supplemented with AA in the diets of summer flounder (*Paralichthys dentatus*) [69] and blunt-snout bream (*Megalobrama amblycephala*) [44], respectively, did not change the whole-body chemical composition. In contrast, the whole-body composition of red hybrid tilapia was noticeably altered by dietary replacement of FM with CPC fortified with lysine [10]. In addition, diets substituting FM with various plant-based protein sources led to remarkable differences in the chemical composition of shortfin corvine but did not in red drum [17]. Nandakumar et al. [55] also found that FM replacement with CGM in diets influenced the whole-body composition of Asian seabass (*Lates calcarifer*). The inconsistency in the results could be explained by a variety of factors, such as the specific fish species and their developmental stage, dietary composition and nutritional profile, the origin and quality of the feed ingredients tested, and the experimental conditions [30].

The AA profiles of the whole-body rockfish were not remarkably impacted by dietary FM replacement with CPC in this experiment. Likewise, a recent investigation conducted by Baek et al. [11] revealed that the AA profiles of the whole-body olive flounder remained unaffected when 25 and 50% FM were substituted with different plant protein sources, including CGM, SPC, and CPC, in diets that included jack mackerel meal. Also, substitution of different plant and animal protein sources for FM in diets did not change the AA profiles of Nile tilapia [70]. Contrary to this experiment, however, dietary FM substitution with SPC changed AA profiles of olive flounder [43] and pearl gentian grouper (*Epinephelus lanceolatus* ♂ × *E. fuscoguttatus* ♀) [71].

Plasma parameters are widely regarded as common biomarkers of fish health and are increasingly used to assess the effects of dietary nutrients and/or their interactions on physiological changes and overall health of fish [72–74]. None of the plasma parameters of rockfish were noticeably influenced by dietary treatments in this study, suggesting that dietary FM replacement with CPC did not detrimentally change the plasma conditions of rockfish. Likewise, Seong et al. [64] demonstrated no discernible changes in the blood chemistry of olive flounder when FM was replaced with fermented plant protein concentrate in the diet. However, unlike this study, Shekarabi et al. [19] found that the plasma parameters of rainbow trout, including ALT, AST, and lactate dehydrogenase, were significantly altered by dietary FM replacement with CPC. Further research on plasma parameters is needed to delve deeper into the potential alterations that may happen when fish are provided with diets replacing alternative protein sources for FM.

The immune system of fish sensitive to plant-origin proteins may be adversely affected by the nutritional imbalance in diets caused by FM replacement [63]. Lysozyme, a mucolytic enzyme, aids in defending fish against microorganisms [75], while SOD acts as an antioxidant enzyme that safeguards fish against harmful oxidative agents, which may negatively affect or damage them [76]. No considerable alterations in SOD and lysozyme activity of rockfish were observed in this experiment, probably implying that FM substitution with CPC in diets had no deleterious effects on these parameters in rockfish. Similarly, dietary substitution of various plant protein sources for FM resulted in no remarkable differences in SOD and lysozyme activity of olive flounder [11,64]. In contrast to the present investigation, Li et al. [77] reported that FM substitution with SPC markedly affected SOD and lysozyme activity in starry flounder (*Platichthys stellatus*). Likewise, serum lysozyme activity in rainbow trout elevated with increased dietary FM replacement with CPC [19]. Another study by Minjarez-Osorio et al. [17] revealed that dietary substitutions of SBM, SPC, and CPC for FM changed the serum lysozyme activity of shortfin corvina. There have been suggestions that these contradictory studies could be clarified by varying levels of tolerance among fish species for plant-derived feedstuffs [17], and dose-dependent influences on the immunity of fish [78].

Replacing FM with plant ingredients in aquafeed is recognized as an environmentally sustainable approach, which diminishes reliance on limited marine fish resources, specifically forage fish stocks used in FM production [5,79,80]. Utilizing plant protein sources like CPC can potentially decrease the aquaculture industry’s reliance on marine fish resources, thereby contributing to the conservation of marine ecosystems and biodiversity. Nevertheless, the nutritional needs of specific fish species may restrict the extent to which FM can be substituted, as EAA in plant-derived ingredients can be inconsistent or unbalanced [81]. Considering the limiting EAAs, particularly lysine, in the CPC-substituted diets could enhance the substitutability of CPC for FM. This study demonstrated that replacing FM up to 10% and 20% with CPC in the 55% FM-based diet of rockfish did not negatively impact the WG and FC, and SGR and FE, respectively.

## Conclusion

The WG and FC, and SGR and FE of rockfish fed the CPC_10_, and CPC20 diets, respectively, were comparable to those of rockfish fed the Con diet. However, the biological indices except for HSI, proximate composition, AA profiles, and plasma and serum parameters of fish were unaffected by dietary FM replacement with CPC. Thus, up to 10% and 20% of FM can be substituted with CPC in the rockfish diet without adversely impacting WG and FC, and SGR and FE, respectively. A further study is recommended to optimize CPC inclusion levels, along with an incorporated palatability enhancer, which could potentially increase FM replacement levels in the rockfish diet.

## Supporting information

S1 Data Growth performance of Rockfish-R1 (XLSX)
